# Nonelderly Adult Cancer Survivors in High Deductible Health Plan: Healthcare Expenditure, Utilization and Access

**DOI:** 10.3390/healthcare9091090

**Published:** 2021-08-24

**Authors:** Ruchira Mahashabde, Chenghui Li

**Affiliations:** Division of Pharmaceutical Evaluation and Policy, Department of Pharmacy Practice, College of Pharmacy, University of Arkansas for Medical Sciences, Little Rock, AR 72205, USA; RVMahashabde@uams.edu

**Keywords:** high deductible plan, health savings account, cancer survivors, access, cost, utilization

## Abstract

**Background**: To compare healthcare expenditure, utilization and access between nonelderly adult cancer survivors enrolled in a high deductible health plan with a health savings account (“HDHP+HSA”), HDHP without HSA (“HDHP alone”) and low deductible health plan (“LDHP”). **Methods**: 1735 cancer survivors, aged 18–64 years, with continuous private coverage identified from the 2012–2017 Medical Expenditure Panel Survey: HDHP alone (n = 353), HDHP+HSA (n = 242) and LDHP (n = 1140). Healthcare expenditures, utilization and inability/delay obtaining medical care were analyzed using generalized linear regressions with inverse propensity score weighting and doubly robust estimation. **Results**: HDHP alone group (23,255 USD) had significantly higher total healthcare expenditure compared to HDHP+HSA (15,580 USD, *p* = 0.012) and LDHP (16,261 USD, *p* = 0.016). HDHP alone (6089 USD; *p* = 0.002) and HDHP+HSA (5743 USD; *p* = 0.012) groups had significantly higher out-of-pocket (OOP) expenditure compared to LDHP (4853 USD). HDHP alone (17,128 USD, *p* = 0.010) and LDHP (12,645 USD, *p* = 0.045) had significantly higher private insurer payments compared to HDHP+HSA (9216 USD). No differences were found in utilization or inability/delay obtaining medical care across groups. **Conclusions**: Non-elderly adult cancer survivors with continuous coverage and comparable sociodemographic characteristics enrolled in HDHP with HSA displayed the lowest healthcare costs compared to HDHP without HSA and LDHP. HDHP+HSA had a significantly higher OOP expenditure than LDHP. No significant differences were observed in utilization or access among groups.

## 1. Introduction

High deductible health plans (HDHP) are now the predominant private insurance plans in the US [1]. The Internal Revenue Service (IRS) defines HDHPs as plans that meet a minimum level of deductible, which changes over time to adjust for inflation of medical costs [2]. In 2020, this level was set at 1400 USD for single coverage and 2800 USD for family coverage. Designed to evoke cost-conscious utilization of healthcare among consumers [3], HDHPs have been associated with reduced healthcare costs and service utilization [4]. An experiment conducted by RAND showed that health insurance plans with higher cost-sharing decreased the use of necessary care in addition to unnecessary care [5]. Further research has shown that enrollees who switched to HDHPs experienced a decline in outpatient visits [6] and emergency department usage [7] as compared to traditional plan enrollees. Some studies have also raised concerns about delayed and forgone care [8] and high financial burdens [9] on families with chronic conditions enrolled in HDHPs.

HDHPs meeting the IRS criteria can be paired with a health savings account (HSA) that offers tax-subsidized savings for medical expenditures [10]. This account is offered mostly by employers, to which both employees and their employers can contribute, although employers are not obligated to offer such an account or contribute to it [11]. Acting as an incentive, the availability of HSA has accelerated the provision and uptake of HDHP by employers. In 2019, 28% of employers in the US offered HDHP, covering 30% of all employees [1]. Large-scale studies have reported reductions in total spending by HDHP enrollees with HSA compared to traditional low deductible health plans (LDHP) [11,12]. However, studies of individual firms found mixed results, reporting lower spending [13], no effect [14] and higher total spending [15]. A review study concluded that though most of the reduction in spending was concentrated among healthy low-risk enrollees, medium-risk enrollees may also show some spending decline [16].

The number of cancer survivors in the United States in 2019 was greater than 16.9 million [17]. Due to advancements in the treatment and diagnosis of cancer, an increase to 20 million survivors is projected by the year 2026 [17]. When compared to the non-cancer population, cancer survivors are known to face higher out-of-pocket costs [18,19]; due to financial challenges, they also experience delayed or forgone care [20], altering of prescription drug usage patterns [21] and medication non-adherence [22]. Another study concluded that difficulty accessing treatment and medication due to cost is a major concern for cancer survivors in the US [23]. HDHP enrollment was found to have similar adverse effects, such as higher out-of-pocket costs [24] and delayed care among women diagnosed with breast cancer [25,26] compared to those without cancer.

There is limited evidence on the effect of HDHP with HSA in cancer survivors. To the best of our knowledge, only three studies have analyzed the effect of HDHP with HSA on cancer survivors [27,28,29]. All three used data from the National Health Interview Survey and studied self-reported financial hardship [27], cost-related medication non-adherence and coping strategies [28], delayed or forgone medical care [28,29] and emergency department (ED) visits [29]. In general, one study concluded that adult cancer survivors aged 18–64 years enrolled in HDHP with HSA have similar experiences as LDHP enrollees [29], while the other two studies concluded that HDHP enrollees without HSA had a higher risk of reported access issues [27,28]. However, to the best of our knowledge, no studies have assessed the effects of HDHP with HSA on healthcare expenditure and health service utilizations other than ED visits in cancer survivors. Assessment of these effects is important because medical care costs, especially costs of cancer-related health services, have significantly increased over the years [30]. Analyzing other health services utilization in addition to ED visits provides a more comprehensive assessment of the effect of HDHP with and without HSA on cancer survivors.

In this nationally representative study of privately insured adult cancer survivors aged 18–64 years, we compared health care expenditure paid by private insurers and survivors/families as well as utilization of various types of health services between enrollees in HDHP with HSA, HDHP without HSA and LDHP. To facilitate comparison with previous studies, we also compared self-reported inability or delay in obtaining medical care or prescription medications across the three groups. We hypothesize that HDHP with HSA will have lower costs and utilization and greater access to care as compared to HDHP, but comparable utilization and costs with LDHP.

## 2. Materials and Methods

### 2.1. Data Source

Privately insured cancer survivors between the ages of 18 and 64 were identified from the 2012–2017 Medical Expenditure Panel Survey (MEPS). MEPS is a nationally representative survey of the civilian non-institutionalized population of the United States [31]. Administered by the Agency of Healthcare Research and Quality, MEPS uses “a stratified, multistage, probability cluster sampling design” and provides survey data on demographic characteristics, health status, insurance coverage, and health service utilization and costs for all individuals in sampled households” [31]. Although the 2018 data are now available to the public, we could not use them for this study due to changes in the survey questions related to the secondary outcome measures.

### 2.2. Study Sample

Cancer survivors were defined as respondents who reported to have ever been diagnosed with cancer or any kind of malignancy. The inclusion criteria were: (1) self-reported cancer diagnosis; (2) being between the ages of 18 and 64 at the end of a year; and (3) having continuous annual private insurance coverage. The exclusion criteria were: (1) self-reported diagnosis of only non-melanoma skin cancer or skin cancer of unknown type; (2) having only Medigap or other than physician/hospital insurance coverage; (3) being covered by multiple private insurance plans; or (4) missing information on deductible, HSA status, out-of-pocket premium or outcome measures. Since Medicare is usually the primary insurer for individuals 65 years and older, their healthcare utilization and costs are likely to be different than those below 65 years and privately insured. Hence, individuals 65 years or older were excluded from the analysis. Details about private insurance coverage, including deductible levels and HSA status, were obtained from MEPS’ Person Round Plan (PRPL) files. These files contain information about the private health insurance plans of participants and link them to the jobs providing insurance [31]. In MEPS, a plan with a deductible greater than or equal to the annually determined IRS (Internal Revenue Service) limit is classified as HDHP. The minimum annual deductible limits for single/family coverage were set at 1200/2400 USD in 2012, 1250/2500 USD in 2013, 1300/2600 USD from 2014 to 2016 and 1350/2700 USD in 2017. Based on the annual deductible and the HSA status, we classified the study sample into three mutually exclusive groups: (1) HDHP without HSA, (2) HDHP with HSA and (3) LDHP.

### 2.3. Outcome Measures

The primary outcome measures included utilization and expenditure for the following services: number of prescription medications, number of office-based visits, number of hospital outpatient visits, any inpatient discharges, and any emergency room visits during a year. We analyzed total healthcare expenditure as the sum of the amount paid by private insurers and that paid out-of-pocket (OOP) by survivors or families (including OOP insurance premium payments) for the above-mentioned services. OOP insurance premium payments were from the PRPL files. Details of health services utilization and expenditure for these services were from the MEPS’ Full Year Consolidated (FYC) files. Consumer Price Index for All Urban Consumers was used to adjust all expenditures for inflation since IRS deductible thresholds were adjusted using these indices [32]. To improve the quality of reporting, upon completion of the household interview and after obtaining permission from the household survey respondents, the MEPS contacts a sample of medical providers by telephone to obtain information that household respondents cannot accurately provide, including dates of visits, diagnosis and procedure codes, charges and payments [31]. For pharmacy use, it collects drug detail information, including the National Drug Code and medicine name, as well as date filled and sources and amounts of payment [31]. This information was used as an imputation source to supplement/replace household reported expenditure information [31]. Due to the nature of the MEPS data, cost and utilization of cancer-related care such as chemotherapy and other anti-cancer treatments were included as a part of total utilization and costs and were not separately analyzed.

The secondary outcome measures were inability to obtain medical care or delay obtaining medical care. They were defined based on an affirmative answer to questions asking “if [person] was unable to receive treatment/prescription medication” or “if [person] had a delay in receiving treatment/prescription medication”. These measures were analyzed separately. 

### 2.4. Covariates

The selection of covariates for the study was guided by Aday and Anderson’s framework on access to care, which identified individual determinants of health services utilization [33]. Based on this framework, the following covariates were included: predisposing factors (age groups (18–39, 40–49, 50–64) [29], sex (male, female), race/ethnicity (Hispanic, non-Hispanic White, non-Hispanic Black, Asian/other), education (≤high school, >high school), marital status (married, not married), region (Northeast, Midwest, West, South)), enabling factors (family income as % of the federal poverty level (low income: <200%, middle income: 200% to 400%, high income: ≥400%), type of insurance coverage (employer-sponsored, other), any public coverage during the year (yes, no)) and need factor (number of chronic conditions (0, 1, 2, ≥3)).

### 2.5. Statistical Analysis

Covariates were compared across the three groups using chi-square tests for all categorical variables.

To adjust for differences in individual characteristics across enrollees in the three types of plans, we conducted a doubly robust (DR) estimation combining a three-way inverse propensity score weighting and regression adjustment of the outcomes. DR estimators are more robust in case of misclassification if either the propensity score model or the outcome model is wrongly specified [34]. The inclusion of additional covariates reduced not only the potential bias but also the standard error of the treatment effect in case some of the covariates were strongly related to the outcome [34].

Propensity scores were estimated using generalized boosted models (GBM), which is a machine learning method that estimates propensity scores through an “iterative process with multiple regression trees”, thus capturing complex and non-linear relationships between variables and leading to the best balance between the treatment and comparison groups [35]. The Toolkit for Weighting and Analysis of Nonequivalent Groups (TWANG) package in the statistical software R was used to handle the three treatment groups using the multinomial propensity scores (MNPS) function [35]. SAS macros for the TWANG package developed by the RAND corporation were used to call functions from the R environment into the SAS environment for conducting the statistical analysis [36].

Calculated propensity scores were then used to weight the three treatment groups to estimate the population average treatment effect. Balance in covariates was assessed before and after weighting. Absolute standardized mean differences (ASMD) <0.1 were regarded as non-significant differences [34]. After a successful balance in covariates was achieved, we estimated the average treatment effects through DR estimation by additional covariate adjustment.

Since both expenditure and utilization measures are non-negative and highly skewed, we applied generalized linear models to compare the differences in healthcare expenditures (with log-link function and gamma distribution), the number of prescription drugs/office-based visits/hospital outpatient visits (with log-link function and negative binomial distribution) and any inpatient discharges/any ER visits/inability or delay in obtaining necessary medical care or prescription medications (with logit-link function and binomial distribution) by adjusting for insurance plan groups and covariates. All models were inversely weighted by estimated propensity scores and survey weights to generate nationally representative estimates.

All analysis was conducted using the SAS, Version 9.4 (SAS Institute, Cary, NC, USA) and Stata/SE 16 (StataCorp, College Station, TX 77845 USA). Statistical significance was determined at α ≤ 0.05 for two-sided tests.

## 3. Results

### 3.1. Sample Size

**Figure 1:**
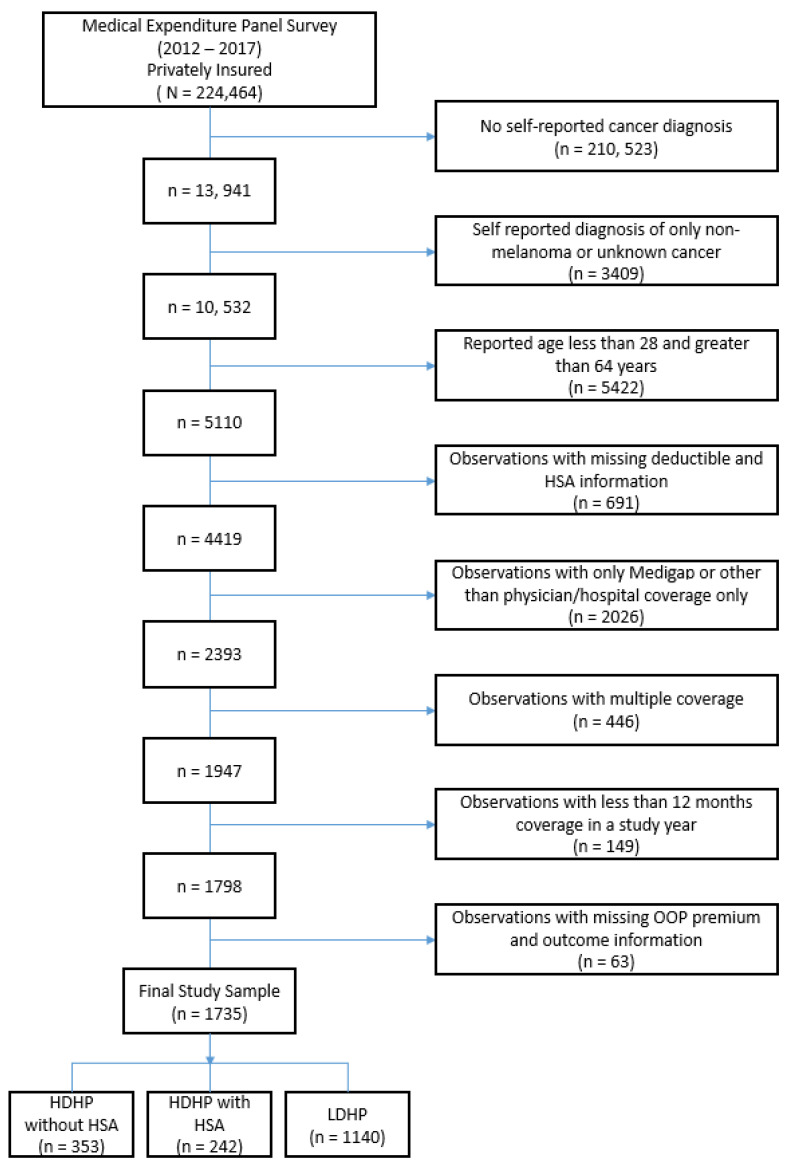
Study sample selection flow chart.

Figure 1 shows the study sample selection flow diagram following the inclusion and exclusion criteria. From 2012 to 2017, 1735 observations of privately insured cancer survivors 18–64 years of age were identified. The final sample was divided into three mutually exclusive groups: HDHP without HSA (n = 353), HDHP with HSA (n = 242) and LDHP (n = 1140).

### 3.2. Individual Characteristics

Table 1 compares the individual characteristics across the groups before and after weighting. Before weighting, cancer survivors in the LDHP group had higher proportions of Hispanics and Non-Hispanic Blacks and were less likely to be in the Midwest region than those in the HDHP with or without HSA groups. Cancer survivors in the HDHP with HSA group were more likely to have greater than high school education and high income than those in the HDHP without HSA or LDHP groups. Compared to other groups, cancer survivors in the LDHP group were less likely to have employer-sponsored plans.

After weighting, there were no statistically significant differences across the groups. The absolute standardized mean differences (ASMD) of pairwise comparisons across the three groups before and after weighting are also shown in Table 1. After weighting, all ASMD was less than 0.1 except the indicator variable for any public coverage during the year (ASMD = 0.114). In the DR estimation, the regression analysis adjusted for all covariates, including this variable.

### 3.3. Outcome Measures

Results for all the outcome measures from the generalized linear models using DR estimation are displayed in Table 2.

### 3.4. Healthcare Expenditure

Among the study groups, the predicted average annual total healthcare expenditure was significantly higher in HDHP without HSA (23,255 USD), compared to HDHP with HSA (15,580 USD, *p* = 0.012) and LDHP (16,261 USD, *p* = 0.016). No significant differences were observed between HDHP with HSA and LDHP (*p* = 0.681). The predicted average annual out-of-pocket healthcare expenditure showed a similar trend with HDHP without HSA, having the highest expenditure of 6089 USD, followed by HDHP with HSA with 5743 USD and LDHP with 4853 USD. HDHP without HSA (*p* = 0.002) and HDHP with HSA (*p* = 0.012) were both significantly higher than LDHP. However, there was no statistically significant difference between HDHP without HSA and HDHP with HSA (*p* = 0.464). Comparison of healthcare expenditure by private insurers showed that both HDHP without HSA (17,128 USD, *p* = 0.010) and LDHP (12,645 USD, *p* = 0.045) had significantly higher predicted average annual payments when compared to HDHP with HSA (9216 USD). No significant differences were found between HDHP without HSA and LDHP (*p* = 0.137).

### 3.5. Health Services Utilization

HDHP without HSA was found to have 19.88 mean predicted annual prescription medications as compared to 18.42 in HDHP with HSA (*p* = 0.526) and 17.30 for LDHP (*p* = 0.166). HDHP without HSA also displayed 11.24 mean predicted annual office-based visits (OB) and 1.70 mean predicted annual outpatient (OP) visits as compared to HDHP with HSA (OB = 10.92, *p* = 0.795 and OP = 1.30, *p* = 0.225) and LDHP (OB = 10.03, *p* = 0.174 and OP = 1.34, *p* = 0.227). However, none of these associations were found to be statistically significant. The predicted proportion of any inpatient discharges (IP) and any emergency room visits (ER) in HDHP without HSA was 13.07% and 18.65%, respectively. The predicted proportions of the aforementioned healthcare services in HDHP with HSA (IP = 11.25%, *p* = 0.564 and ER = 17.62%, *p* = 0.778) and LDHP (IP = 10.84%, *p* = 0.336 and ER = 15.79%, *p* = 0.303) were also not found to be significantly different when compared to HDHP without HSA.

### 3.6. Inability/Delay in Obtaining Medical Care

Both HDHP without HSA (6.40%, *p* = 0.185) and HDHP with HSA (4.48%, *p* = 0.751) had a slightly higher (but statistically no significant) probability of reporting inability in obtaining medical care compared to LDHP (3.91%). Delay in obtaining medical care showed a higher (and non-significant) trend in HDHP with HSA (10.93%) compared to HDHP without HSA (8.09%, *p* = 0.365) and LDHP (8.75%, *p* = 0.425).

## 4. Discussion

In this study of adult cancer survivors aged 18–64 years who were continuously enrolled in a private insurance plan, we observed that having HSA with HDHP affected healthcare expenditures. Total expenditure was highest in HDHP alone without HSA, and it was comparable between HDHP with HSA and LDHP groups. OOP expenditure was lower in HDHP with the HSA group compared to HDHP without HSA but was still higher than LDHP. Healthcare expenditure by private insurers was lowest in HDHP with the HSA group. Utilization of healthcare services, however, was found to be similar across the three groups. Additionally, there was no statistically significant difference in reported inability or delay in obtaining medical care or prescription medication across the three groups. To our knowledge, this is the first nationally representative study that has assessed the association of an HSA with HDHP with total and OOP healthcare expenditure among cancer survivors. Our findings are important considering significant increases in HDHPs enrollment (from 4.2% in 2007 to 18.9% in 2017) [37] and costs of cancer-related health services in the US [30]. Furthermore, through the analysis of the utilization of other health services in addition to ED visits, we provide a more comprehensive assessment of HDHPs’ effects on cancer survivors with and without HSA compared to the current literature.

### 4.1. Health Care Expenditures

To the best of our knowledge, no study analyzing healthcare costs in cancer survivors with HSA exists. One study, centering on women undergoing active metastatic breast cancer treatment, reported that women with HDHP without HSA paid higher out-of-pocket costs compared to those with LDHP [24]. Our study results confirmed this finding. In addition, we found total health care costs to be lowest in HDHP with the HSA group compared to HDHP alone or LDHP groups. Previous literature showed that spending reductions associated with HSA were mostly seen among the healthy, low-to-medium-risk population [11,16]. Our study adds to this literature by showing that having an HSA with HDHP may reduce health care costs even among inherently high-risk, high-cost individuals such as cancer survivors without significantly impacting health care utilization.

We additionally found that private insurer expenditure was the lowest for HDHP with the HSA group. If confirmed, this suggests that employers might consider offering and investing in HSA with HDHP for potential cost savings. Only 41% of the high-deductible plan enrollees in our study (HDHP with and without HSA combined) had an associated HSA, leaving a majority with possible HSA enrollment opportunities.

### 4.2. Health Care Utilization

We observed similar utilization of prescription medications, office-based visits, hospital outpatient visits, inpatient discharges and ED visits among the three study groups. Focusing solely on ED visits, one other study also reported similar rates of utilization among cancer survivors who enrolled in the three types of plans using another nationally representative survey [29]. To the best of our knowledge, no other studies have analyzed the effect of HDHP and HSA status on cancer survivors’ utilization of health care services other than EDs.

High deductibles were introduced with the intent to evoke “cost-conscious” behavior among enrollees, ultimately reducing unnecessary healthcare utilization [38]. However, during the course of their necessary treatments, recently diagnosed or recurrent cancer survivors are likely to quickly exceed the annual deductible limits and reach OOP maximums. Thus, “cost-conscious” behavior and the subsequent reduction in utilized services among cancer survivors enrolled in HDHP with or without HSA might not be possible.

### 4.3. Inability/Delay Obtaining Medical Care

We are aware of only one other study concerning HSA status that analyzes access to care in a nationally representative cancer survivor population. It concluded that cancer survivors enrolled in HDHP without HSA were more likely to have delayed or forgone care compared to those with HDHP with HSA or LDHP [29]. On the contrary, our study found no significant difference in the inability or delay of obtaining treatment and prescription medication across the three groups. Apart from having differently defined survey questions for the access variables, we also restricted our study population to those enrolled in one private plan for an entire year. The reference study allowed multiple coverages, and whether a person was continuously covered in the whole year was unknown. Our choice allowed us to analyze a plan’s effect without the possible influence of coverage gaps and/or having multiple plans simultaneously. Thus, our study uniquely analyzes the association of continuous annual private health insurance coverage with health care utilization, costs and access. In addition, compared to the previous study sample, our study sample had a higher proportion of cancer survivors with high school or less education and more comorbidities, both of which can increase health services utilization in all groups [39], which may lead to fewer differences across groups.

## 5. Limitations

This study has several limitations. Being a cross-sectional observational study, it cannot establish causality of plan type on the outcomes. Survivor cancer histories and outcome measures were self-reported and might be prone to recall bias. Detailed cancer histories and time since cancer diagnosis information were not available in MEPS data. Information about plan-specific deductible amounts and employer contributions to HSA was not available, possibly obscuring patterns of enrollee behavior. Data concerning reasons for plan enrollment were unavailable. However, we controlled for important socioeconomic factors and comorbidities through DR estimation, which combines inverse propensity score weighting and regression adjustment, thus diminishing the possibility of selection bias. In generating the inverse propensity score weights, we noticed that the weighted groups have distributions of baseline characteristics more similar to HDHP with the HSA group. Thus, the interpretation of our results may be more aligned with the “counterfactual” outcomes if the HDHP with HSA enrollees are instead enrolled in HDHP without HSA or LDHP. Although Medicare is the primary insurer for the US population 65 or older, a small proportion of elderly individuals continue to have private insurance coverage as their primary coverage through their employers. In our data set, out of the 884 cancer survivors 65 or older, 97% had both Medicare and private insurance coverage. For an individual to contribute to an HSA, one cannot have any health insurance other than HDHPs [40]. Individuals who are dually enrolled may keep the HSA but cannot contribute to it and are likely to have different health care utilization or costs from those under 65 [41]. In our data set, only 26 of the 884 cancer survivors 65 or older had private insurance as their insurance coverage without Medicare enrollment during the study year. Due to their small sample size, they were excluded from our analysis. Nonetheless, elderly cancer survivors 65 or older represent a very important group of cancer survivors [42]. The impact of high deductible and HSA on the healthcare utilization and costs among cancer survivors who continue to have private insurance coverage beyond 65 without Medicare enrollment should be studied in larger databases further.

## 6. Conclusions

In this nationally representative study of non-elderly adult cancer survivors aged 18–64 years, we found HDHP with HSA had the lowest total health care costs compared to HDHP without HSA and LDHP. However, no significant differences were observed in the utilization of healthcare services or reported inability/delay in obtaining medical care among the three groups. Future research with larger samples incorporating health insurance plan specifics and additional cancer characteristics should be conducted to confirm our findings.

## Figures and Tables

**Table 1:** Comparison of individual characteristics across groups before and after weighting.

	Unweighted Distribution				Absolute Standardized Mean Difference (Unweighted)			Weighted Distribution				Absolute Standardized Mean Difference (Weighted)		
	HDHP without HSA	HDHP with HSA	LDHP		HDHP without HSA vs. HDHP with HSA	HDHP without HSA vs. LDHP	HDHP with HSA vs. LDHP	HDHP without HSA	HDHP with HSA	LDHP		HDHP without HSA vs. HDHP with HSA	HDHP without HSA vs. LDHP	HDHP with HSA vs. LDHP
	(n = 353)	(n = 242)	(n= 1140)					(n = 353)	(n = 242)	(n = 1140)				
Variables	%	%	%	*p* ^a^	St. Diff	St. Diff	St. Diff	%	%	%	*p* ^a^	St. Diff	St. Diff	St. Diff
**Age**														
18–39	10.20	15.29	13.86	0.254	0.146	0.105	0.041	10.46	12.95	12.85	0.830	0.072	0.069	0.003
40–49	18.98	20.25	17.54		0.034	0.038	0.072	16.02	17.95	16.85		0.051	0.022	0.029
50–64	70.82	64.46	68.60		0.137	0.048	0.089	73.52	69.09	70.30		0.095	0.069	0.026
**Gender**														
Male	32.01	33.88	33.86	<0.001 *	0.039	0.038	0.001	36.07	32.88	36.40	0.657	0.066	0.007	0.073
Female	67.99	66.12	66.14		0.039	0.038	0.001	63.93	67.12	63.60		0.066	0.007	0.073
**Race/Ethnicity**														
Hispanic	9.35	7.44	15.44	<0.001 *	0.071	0.226	0.297	5.65	6.10	7.52	0.954	0.017	0.069	0.053
Non-Hispanic White only	75.35	79.75	61.67		0.109	0.339	0.448	83.05	83.04	80.60		0.000	0.061	0.061
Non-Hispanic Black only	8.22	7.02	15.79		0.045	0.285	0.330	6.67	6.22	7.19		0.017	0.020	0.036
Others	7.08	5.79	7.11		0.060	0.001	0.061	4.63	4.64	4.70		0.001	0.003	0.003
**Education Level**														
Highschool or less than high school	50.99	35.95	49.39	<0.001 *	0.302	0.032	0.270	46.88	42.84	45.63	0.675	0.081	0.025	0.056
Greater than high school	49.01	64.05	50.61		0.302	0.032	0.270	53.12	57.16	54.37		0.081	0.025	0.056
**Marital Status**														
Not married/missing	26.35	35.12	34.12	0.017 *	0.190	0.168	0.022	27.63	29.79	29.2	0.864	0.047	0.034	0.013
Married	73.65	64.88	65.88		0.190	0.168	0.022	72.37	70.21	70.8		0.047	0.034	0.013
**Region**														
North East	13.03	13.22	17.63	<0.001 *	0.005	0.119	0.114	15.60	15.39	16.79	0.992	0.005	0.031	0.036
Midwest	25.78	30.58	19.12		0.116	0.160	0.276	24.56	23.02	22.55		0.037	0.048	0.011
South	38.53	30.99	34.56		0.160	0.084	0.076	36.38	36.35	34.59		0.001	0.038	0.037
West	22.66	25.21	28.68		0.058	0.136	0.079	23.46	25.24	26.07		0.040	0.059	0.019
**Family income as % of federal poverty level**														
Low income or less or missing (<200%)	16.43	7.44	14.39	0.007 *	0.295	0.067	0.228	10.79	8.44	10.89	0.876	0.077	0.003	0.080
Middle income (200%–400%)	28.05	25.62	29.04		0.057	0.023	0.081	24.11	24.23	23.32		0.003	0.019	0.021
High income (≥ 400%)	55.52	66.94	56.58		0.241	0.022	0.219	65.10	67.33	65.79		0.047	0.015	0.033
**Type of insurance coverage**														
Other	19.55	7.44	5.26	<0.001 *	0.506	0.596	0.091	9.15	7.37	7.57	0.699	0.074	0.066	0.008
Employer-sponsored	80.45	92.56	94.74		0.506	0.596	0.091	90.85	92.63	92.43		0.074	0.066	0.008
**Any public coverage**														
No	92.92	95.45	90.35	0.021 *	0.095	0.096	0.191	93.97	95.81	92.75	0.310	0.069	0.046	0.114
Yes	7.08	4.55	9.65		0.095	0.096	0.191	6.030	4.19	7.25		0.069	0.046	0.114
**Comorbidities**														
0	19.26	18.6	18.07	0.379	0.018	0.032	0.014	16.19	18.34	16.97	0.996	0.057	0.021	0.036
1	20.96	27.27	20.61		0.155	0.009	0.164	19.71	20.34	20.31		0.015	0.015	0.001
2	20.68	19.01	22.37		0.039	0.040	0.079	23.47	22.97	23.63		0.012	0.004	0.015
>3	39.09	35.12	38.95		0.082	0.003	0.079	40.63	38.35	39.09		0.047	0.032	0.015
**Year**														
2012	17.28	14.88	21.40	<0.001 *	0.062	0.107	0.169	18.19	18.61	18.21	1.000	0.011	0.001	0.010
2013	15.01	10.74	17.89		0.115	0.077	0.192	15.26	15.97	16.13		0.019	0.024	0.004
2014	15.58	17.36	15.96		0.048	0.010	0.037	16.20	15.27	16.79		0.025	0.016	0.041
2015	14.45	15.70	16.49		0.033	0.054	0.021	17.52	16.91	16.68		0.016	0.022	0.006
2016	18.70	19.83	16.40		0.030	0.061	0.091	18.30	18.52	17.73		0.006	0.015	0.021
2017	18.98	21.49	11.84		0.073	0.207	0.279	14.54	14.72	14.45		0.005	0.003	0.008

^a.^*p*-values are from chi-square test. * Values significant at α ≤ 0.05. HDHP without HSA = high deductible health plan. HDHP with HSA = high deductible health plan with health savings account. LDHP = low deductible health plan.

**Table 2:** Results from the Generalized Linear Models using Double Robust (DR) Estimation.

Variable				LDHP as Reference	HDHP with HSA as Reference
	Predicted Mean	95% CI		*p* Value	*p* Value
**HEALTHCARE EXPENDITURE ($)**					
**Total**					
HDHP without HSA	$23,255	($17,207–$29,302)		0.016 *	0.012 *
HDHP with HSA	$15,580	($12,772–$18,387)		0.681	
LDHP	$16,261	($13,667–$18,854)			
**Self ($)**					
HDHP without HSA	$6089	($5322–$6857)		0.002 *	0.464
HDHP with HSA	$5743	($5135–$6350)		0.012 *	
LDHP	$4853	($4460–$5245)			
**Private Insurer ($)**					
HDHP without HSA	$17,128	($11,176–$23,080)		0.137	0.010 *
HDHP with HSA	$9216	($6775–$11,657)		0.045 *	
LDHP	$12,654	($9526–$15,782)			
**HEALTHCARE UTILIZATION**					
**Number of Prescription Meds**					
HDHP without HSA	19.88	(16.70–23.06)		0.166	0.526
HDHP with HSA	18.42	(15.21–21.64)		0.552	
LDHP	17.30	(15.14–19.46)			
**Number of Office Based Visits**					
HDHP without HSA	11.24	(9.58–12.90)		0.174	0.795
HDHP with HSA	10.92	(9.19–12.66)		0.978	
LDHP	10.03	(9.08–10.98)			
**Number of Outpatient Visits**					
HDHP without HSA	1.70	(1.13–2.28)		0.227	0.225
HDHP with HSA	1.30	(0.86–1.74)		0.884	
LDHP	1.34	(1.03–1.64)			
**Any Inpatient discharges (%)**					
HDHP without HSA	13.07%	(9.01%–17.13%)		0.336	0.564
HDHP with HSA	11.25%	(6.76%–15.73%)		0.873	
LDHP	10.84%	(8.61%–13.06%)			
**Any Emergency Room visits (%)**					
HDHP without HSA	18.65%	(13.92%–23.39%)		0.303	0.778
HDHP with HSA	17.62%	(12.11%–23.12%)		0.567	
LDHP	15.79%	(12.80%–18.79%)			
**ACCESS TO CARE**					
**Unable to Get Care (%)**					
HDHP without HSA	6.40%	(2.97%–9.82%)		0.185	0.412
HDHP with HSA	4.48%	(1.64%–7.31%)		0.751	
LDHP	3.91%	(2.02%–5.81%)			
**Delay Obtaining Care (%)**					
HDHP without HSA	8.09%	(4.84%–11.34%)		0.743	0.365
HDHP with HSA	10.93%	(6.04%–15.82%)		0.425	
LDHP	8.75%	(6.51%–10.99%)			

* Values significant at α ≤ 0.05. HDHP without HSA = high deductible health plan. HDHP with HSA = high deductible health plan with health savings account. LDHP = low deductible health plan. Total: sum of self and private insurer expenditures. Self: healthcare expenditure and health insurance premiums paid out-of-pocket by survivors or their families. Private Insurer: health care expenditure paid by private insurers. DR estimation applied regression models of health outcome measures adjusting for covariates and inversely weighted by estimated propensity scores and survey weights. Margins were used to generate predicted means across the groups.

## Data Availability

Medical Expenditure Panel Survey is a public use database downloadable from Medical Expenditure Panel Survey Download Data Files https://www.meps.ahrq.gov/mepsweb/data_stats/download_data_files.jsp Accessed: 19 August 2021.
